# An Assessment of Handover Culture and Preferred Information in the Transitions of Care of Elderly Patients

**DOI:** 10.7759/cureus.5267

**Published:** 2019-07-29

**Authors:** Sachin Trivedi, Alixe Dick, Stephanie Beckett, Riley J Hartmann, Christopher Roberts, Kish Lyster, James Stempien

**Affiliations:** 1 Emergency Medicine, University of Saskatchewan, Saskatoon, CAN; 2 Emergency Medicine, University of Saskatchewan, Regina, CAN; 3 Internal Medicine, Regina Qu'appelle Health Region, Regina, CAN

**Keywords:** geriatrics, transitions of care, communication

## Abstract

Introduction

Transitions of care for elderly patients in long term care (LTC) to the emergency department (ED) is fraught with communication challenges. Information preferred during these transitions has not been agreed upon. We sought to understand our local handover culture and identify what information is preferred in the transitions of care of these patients.

Methods

We performed a cross-sectional electronic survey that was distributed to 1470 healthcare providers (HCPs) and 82 patient and family advocates (PFAs) in two Canadian cities. The HCP group consisted of physicians and nurses in ED and LTC settings as well as paramedics. The survey was open for a period of one month with formal reminders sent weekly.

Results

A total of 12.9% of HCPs and 26.8% of PFAs responded to the survey. Only 41.3% of HCP respondents were aware of existing handover protocols and 83.2% indicated a desire for a single page handover form. HCPs identified concerns over handover culture surrounding workplace inefficiencies and increased demands to their time. Several preferred items of information in the transitions of care for the institutionalized elderly patient were also identified across both HCP and PFA groups.

Conclusions

Our study identified a need for improved local handover culture in transitions of care for the institutionalized elderly patient. We also identified the preferred elements of information during bilateral communication between LTC and the ED. Our results will be used to design a patient-centred handover form for future use in this population.

## Introduction

Elderly patients who are institutionalized in long term care (LTC) facilities represent a challenging demographic in the provision of emergency care. These patients may be unable to appropriately communicate their concerns when presenting to the Emergency Department (ED), owing to different disease states which can alter physical or cognitive function. When patients in LTC facilities have an acute medical concern, they are often transferred to the ED for assessment by an emergency physician. At this point, transitional care requires multiple handovers between a variety of healthcare providers (HCPs) including paramedics as well as LTC and ED staffs.

Transitional care has been identified as having risks to patient safety, with adverse events such as medication errors and care delays having been highlighted as common occurrences [1-2]. Communication during handovers has been cited as an area for intervention [3-7]. Previous studies have demonstrated a role for standardized handover forms in the elderly population, but there still appears to be a lack of consensus on what information is necessary to communicate on these forms [4-5,8-9].

It is clear that handovers in the transitional care of this demographic represent an area which should be targeted for quality improvement work. The impetus for improvement in this area is further amplified with literature demonstrating that elderly patients are more frequently using the ED, with patients within LTC settings being found to have more ED-revisits compared to those who are community-dwelling [10-12].

There has been a recent call for the creation of standardized, “receiver-driven” handover protocols [13]. With respect to the LTC elderly population, it has been recommended that standardized communication protocols be implemented with multidisciplinary input [14]. In this project, we performed a survey of key stakeholders involved in the transitions of care of elderly patients between LTC facilities and the ED. Our goal was to understand the underlying handover culture as well to ascertain what information is preferred in this transitional period. The ultimate goal of this project was to garner the required information needed to plan the design and implementation of a handover form for this population.

## Materials and methods

Study setting and ethical considerations

We performed a cross-sectional, electronic survey which was distributed to a total of 1470 HCPs and 82 patient and family advocates (PFAs). In the HCP group, the survey was sent to nurses and physicians in both LTC and ED settings, as well as paramedics. Participants were from two medium-sized, urban Canadian cities in the province of Saskatchewan, either Regina or Saskatoon. In total, the survey was distributed to four EDs, four LTC facilities, two Emergency Medical Services organizations and two PFA councils across the two cities. The LTC facilities selected were those operated by the local regional health authority. We received an ethical exception from the University of Saskatchewan’s Research Ethics Board as this was a Quality Improvement project.

Survey design and recruitment

Survey questions were designed with the intent of understanding the local handover culture as well as identifying essential information in transitional care of the elderly. Questions were created following a literature review on transitional care in the elderly population. Two separate surveys were created, one for each enrolled group. Content between the surveys was largely similar, with questions on handover culture being limited to the HCP survey. In total, 12 questions were created and these were of either multiple choice or free-text entry type (see Appendix A). Participants were recruited through electronic mail. The surveys were either directly sent to participants or through a designated contact person who represented each group.

Data collection and statistical analysis

This survey was administered electronically using the SurveyMonkey© 2018 (SurveyMonkey Canada Inc., Ottawa, ON) platform. An electronic consent form was embedded in the survey platform. Prior to distribution, the entire research team pilot-tested the survey to ensure clarity and functionality. Data collection occurred over a period of one month with formal reminders sent weekly. At the end of the data collection period, data were exported into a Microsoft Excel© 2017 (Microsoft Corp., Redmond, WA) spreadsheet for further analysis. The Social Science Research Laboratory at the University of Saskatchewan assisted us in the statistical analysis of this project. Descriptive statistics are reported. Independent sample t-tests were also performed to assess differences between the HCP and PFA groups. Finally, free-text responses were reviewed qualitatively and grouped according to common themes.

## Results

The overall response rate to our survey was 12.9% (191/1470) of HCPs and 26.8% (22/82) of PFAs participated. Within the HCP group, 38.2% (73/191) were paramedics, 37.6% (72/191) were emergency staff and 24.1% (46/191) worked within LTC.

Handover culture

When participants within the HCP group were asked if their organizations had existing handover protocols, 41.3% (79/191) responded “yes”, 41.3% (79/191) responded “no” and 17.3% (33/191) were uncertain. Those who responded “yes” described the protocols as consisting of a handover form or having the patient’s chart accompany them on transfer between facilities. Participants also indicated that handover protocols varied by the facility. When HCPs were asked if they would find a single page handover form to be useful, 83.2% (159/191) were in favour of this.

Answers to free-text entry questions revealed that there were significant concerns surrounding efficiency. For example, ED staff claimed that they received very little, if any, information when patients were transferred. LTC staff noted that they were overwhelmed with the amount of paperwork they have to fill out and that filling out a handover form requires additional time which they may not have. Some HCP respondents also commented that they felt that patients were transferred to the ED needlessly, especially in cases of advanced care directives stating that patients are not for resuscitation or transfer to hospital.

Preferred information in transitional care

**Figure 1:**
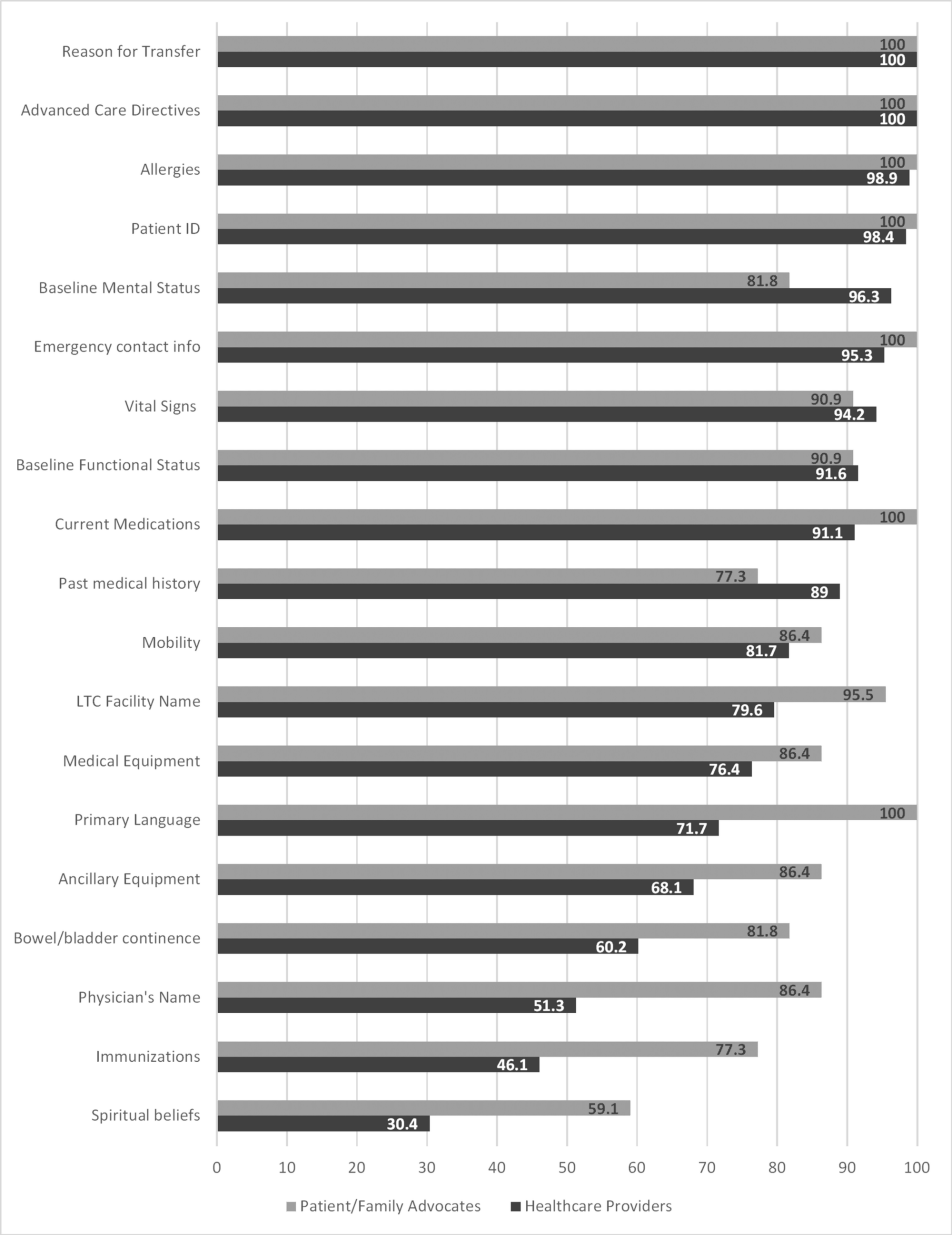
Preferred information to be communicated between long term care facility and emergency care providers

Figure 1 displays the results by the respondent group with respect to what information was felt to be essential to the transitional care of LTC patients as they go from their home facility to the ED. Both groups identified that the most important items of information to include were the reason for transfer, advanced care directives, allergies and patient identification data. Independent sample t-tests revealed that there were some significant differences in scores between the groups for several items. In all of these cases, the PFAs viewed these items as a higher priority to include: primary language (p=0.001), current medications (p=0.001), the name of the patient’s primary care physician (p=0.001), the name of the patient’s LTC facility (p=0.005), immunization status (p=0.004), ancillary equipment (p=0.034), bowel and bladder continence (p=0.002), emergency contact information (p=0.002) and spiritual beliefs (p=.0.007).

**Figure 2:**
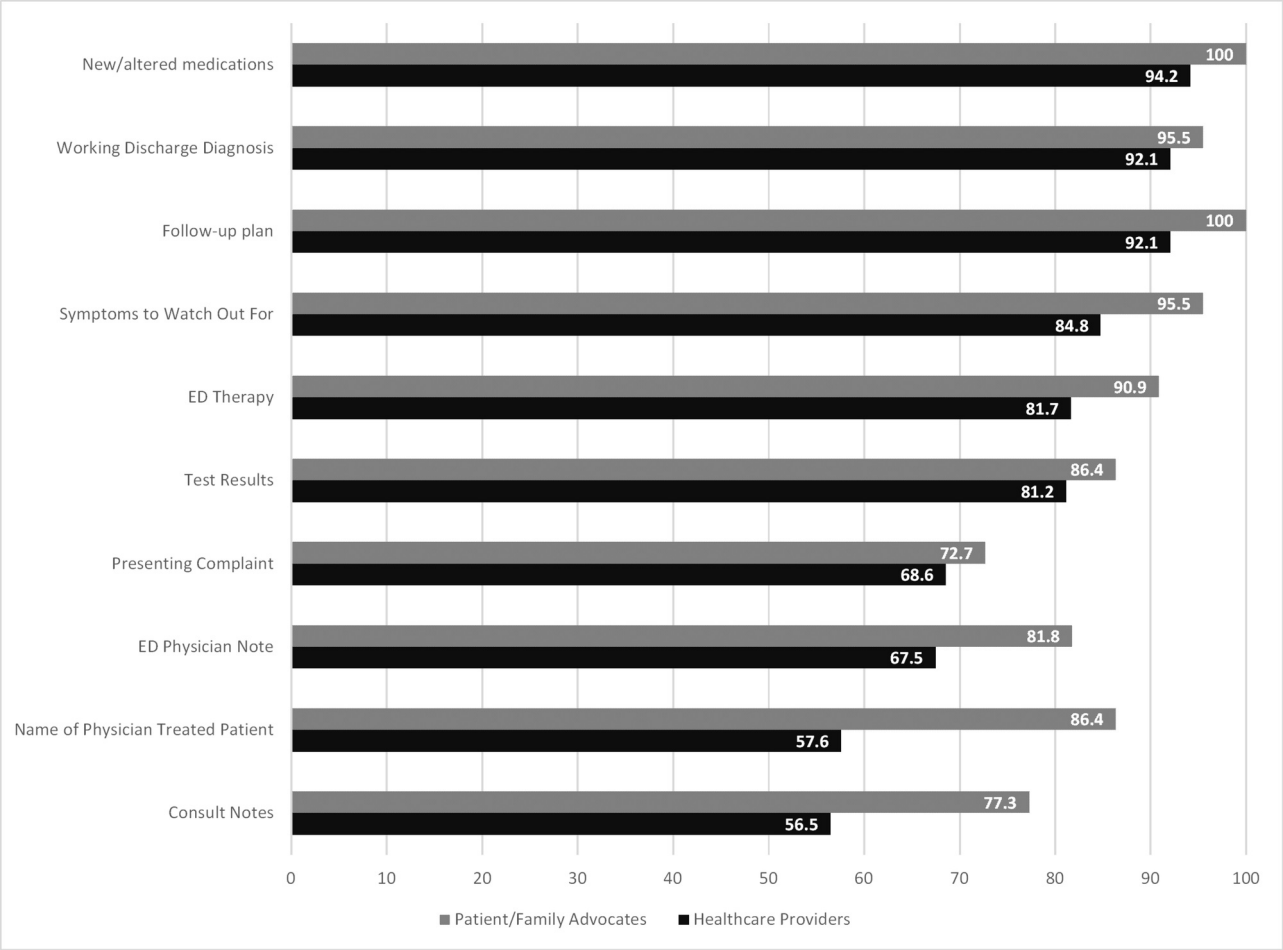
Preferred information to be communicated between emergency care providers and long term care facilities

Figure 2 displays the results by the respondent group with respect to what information was felt to be essential for patients transitioning back to their LTC facility upon discharge from the ED. Again, there were differences noted in the scores between the groups for some items. For transfers back to an LTC facility, PFAs viewed alterations to medications (p=0.001), a documented follow-up plan (p=0.001), and the name of the physician who treated the patient (p=0.002) to be a higher priority.

 

Participants also gave suggestions on what elements would be required to ensure completion of any future handover form. Respondents suggested that a standardized pre-transfer checklist should be implemented, with forms being auto-populated based on other documents. Respondents also highlighted a desire for any handover form to be standardized across all facilities and for the forms to be easy to complete.

## Discussion

Our study is the first which has surveyed both HCPs and PFAs with regards to transitions of care in the elderly population. With respect to handover culture, our results revealed a suboptimal understanding of local procedures, but a strong desire for a more streamlined handover process. In addition to this, HCPs expressed significant concern over inefficiencies in the transition process and being overwhelmed in their current work environment. With respect to the preferred information in transitional care, a representative sample of the HCPs involved in this process outlined the items necessary to include in bilateral communication between the ED and LTC facilities. Furthermore, we have identified items which PFAs have given importance to. Ultimately, our results have provided the foundation from which a patient-centered transition of care handover form can be developed.

In looking at handover culture, we demonstrated that many HCPs were either unaware or uncertain about the existence of formal handover procedures. Though this result has not been as explicitly stated in previous literature, studies have shown that formalized transfer protocols are either not followed, or that transfer forms are either not received by the ED or are incomplete [5-6,8,15]. These results highlight the challenges associated with implementing systemic procedures, but may also reflect a deficiency in organizational safety culture. As such, it may be prudent for organizations to formally educate staff on the importance of transitions of care in order to improve the visibility of this aspect of care. In non-geriatric populations, large scale education on the adoption of formalized handover procedures has been previously demonstrated to be beneficial [16-17]. Additionally, use of evidence-based implementation strategies, such as educational outreaches and interactive training sessions may prove to be beneficial in achieving a positive cultural shift [18].

Our results revealed concerns from HCPs over inefficiencies and being overwhelmed in their work environment. This represents a significant barrier in the successful implementation of formalized handover protocols. Specifically, respondents cited time constraints as a negative influence in completing transfer forms. In the era of the electronic medical record (EMR), it stands that handover procedures would benefit from being engrained within EMR infrastructure. Auto-populated forms and templates have been demonstrated to overcome this challenge and increase end-user engagement and thus merit consideration [17,19].

With respect to identifying the essential information in the transitional care of the elderly, our results have provided the background information needed to implement locally relevant transitions of care handover form. Previous literature has demonstrated the lack of agreement on what information is required, indicating that there may not be a universally applicable solution [4,9]. Though our study now adds to the existing knowledge base, this lack of agreement highlights that local factors should be considered in the design and implementation of any transitions of care intervention. 

To our knowledge, our study is the first which has sought the opinions of an interdisciplinary HCP group as well as PFAs. An interdisciplinary approach and patient involvement have been advocated for in the past but there appears to be a paucity of published literature which has involved PFAs in this area [3,14]. Furthermore, interdisciplinary co-design and implementation of transfer protocols have been demonstrated to improve communication [19]. PFA representation in our study will help to create a handover form which is patient-centred as we have now identified the information that is relevant to all the key stakeholders involved in this population and not just the clinical team.

There were some limitations to our study. As an external survey, we had a low overall response rate which may limit the generalizability of our findings. Generalizability may also be limited in that participants were from two medium-size Canadian cities and so the results and identified challenges may not be applicable to institutions of smaller or larger populations. As well, though the questions were designed with the elderly population in mind they were not explicitly stated as such and so respondents may not have considered the needs of elderly patients when completing the survey. Additionally, recruitment was not standardized. For some surveyed groups, we were reliant on a single contact person external to the research team to forward the recruitment and reminder letters to their representative sample and this may not have been completed as intended. Finally, our research team did not include an expert in geriatric medicine and so our survey questions may have not captured some relevant themes.

## Conclusions

Our study identified that there is a need for an improved handover culture to be established when caring for LTC patients during transitions in and out of the ED. Organizations need to make an active effort to provide education around the importance of transitions of care and the existence of local handover protocols. We also identified preferred information in the transitions of care of these patients according to HCPs and PFAs, including reason for transfer, advanced care directives, allergies and patient identification data. Our findings will be used to generate a patient-centred handover form to be used on bilateral transfers of care between LTC facilities and the ED. Future research will involve piloting of this form and evaluation of its success.

## Appendices

Appendix A. Survey Questions

Q1: Please select your primary language.

English

Spanish

Q2: Please select the city where you live/work or your health region.

Regina (Regina Qu’Appelle Health Region)

Saskatoon (Saskatoon Health Region)

Q3a: (For healthcare providers): Please select your role

Emergency staff (physician, nurse)

Paramedic

Long-term care staff (physician, manager, nursing staff, care aid)

Q3b: (For patient advocates): Please select your role - Patient/family representative

Yes

No

Q4 (For healthcare providers): Does your organization/centre currently have any protocols for handover of information when patients are transferred from Long-term care (LTC) facility to the Emergency Department (ED)?

Yes

No

Q5 (For healthcare providers): If you selected 'yes' to the previous questions, please describe the protocol utilized in your facility. If you selected 'no' to the previous question please choose 'not applicable'.

Chart goes with patient

Hand over form - please describe

Other - please describe

Not Applicable

Q6 (For healthcare providers): Would you find a 1-page standardized handover form helpful?

Yes

Not sure

Q7: What information do you think is essential to be communicated between the staff at a patient’s long-term care facility to an emergency care provider?

Patient ID (name, date of birth)

Primary language spoken

Baseline mental status

Baseline functional status

Vital signs at the time of complaint (ex: heart rate, respiration rate, blood pressure)

Reason for transfer

Current medications

Advanced care directives

Medical equipment (home oxygen, ostomy supplies, CPAP machine)

Primary care physician name

Long-term care facility name, charge nurse, phone number of facility

Allergies

Immunizations

Mobility

Ancillary equipment needs (wheelchair, walker, hearing aids)

Bowel/bladder continence

Past medical and surgical history

Emergency contact information

Spiritual beliefs

Q8: Would it be important to include if the patient was seen by a healthcare professional before the transfer?

Yes

Not sure

Q9: What information do you think needs to be communicated between an emergency care provider and long-term care facility staff when a patient leaves the emergency department to return back to their care facility?

Presenting complaint

Test results and interpretations

ED therapy and clinical response

Consult notes (in person or via telephone) in ED

ED physician note, or copy of dictation

Working discharge diagnosis

New prescription and alterations to long-term medications

Follow-up plan

Name of physician that treated the patient

Reasons to return to the ED (symptoms to watch out for)

Other, please specify

Q10: (Is there anything else you would add to the form?) What information do you think is essential to be communicated between the staff at a patient’s long-term care facility to an emergency care provider?

Q11: What do you see as being most beneficial towards ensuring completion and regular use of the handover form? (For example, instituting a checklist before transfer, or having most of form pre-filled out and kept up to date by LTC physician)

Q12: Please include any additional comments you have.
